# Trends in HIV Prevention, Treatment, and Incidence in a Hyperendemic Area of KwaZulu-Natal, South Africa

**DOI:** 10.1001/jamanetworkopen.2019.14378

**Published:** 2019-11-01

**Authors:** Ayesha B. M. Kharsany, Cherie Cawood, Lara Lewis, Nonhlanhla Yende-Zuma, David Khanyile, Adrian Puren, Savathree Madurai, Cheryl Baxter, Gavin George, Kaymarlin Govender, Sean Beckett, Natasha Samsunder, Carlos Toledo, Kassahun Abere Ayalew, Karidia Diallo, Mary Glenshaw, Amy Herman-Roloff, Eduan Wilkinson, Tulio de Oliveira, Salim S. Abdool Karim, Quarraisha Abdool Karim

**Affiliations:** 1 Centre for the AIDS Programme of Research in South Africa, Doris Duke Medical Research Institute, Nelson R. Mandela School of Medicine, University of KwaZulu-Natal, Durban, South Africa; 2 Epicentre AIDs Risk Management, Cape Town, South Africa; 3 Centre for HIV and STIs, National Institute for Communicable Diseases, National Health Laboratory Service, Johannesburg, South Africa; 4 Global Clinical and Virology Laboratory, Amanzimtoti, South Africa; 5 Health Economics and HIV/AIDS Research Division, University of KwaZulu-Natal, Durban, South Africa; 6 Centers for Disease Control and Prevention, Atlanta, Georgia; 7 KwaZulu-Natal Research Innovation and Sequencing Platform, University of KwaZulu-Natal, Durban, South Africa; 8 Department of Epidemiology, Mailman School of Public Health, Columbia University, New York, New York

## Abstract

**Importance:**

In Africa, the persistently high HIV incidence rate among young women is the major obstacle to achieving the goal of epidemic control.

**Objective:**

To determine trends in coverage of HIV prevention and treatment programs and HIV incidence.

**Design, Setting, and Participants:**

This cohort study consisted of 2 sequential, community-based longitudinal studies performed in the Vulindlela and Greater Edendale area in KwaZulu-Natal, South Africa. Participants enrolled from June 11, 2014, to June 22, 2015 (2014 survey), with a single follow-up visit from June 24, 2016, to April 3, 2017 (2016 cohort), or enrolled from July 8, 2015, to June 7, 2016 (2015 survey), with a single follow-up visit from November 7, 2016, to August 30, 2017 (2017 cohort). Men and women aged 15 to 49 years were enrolled in the 2014 and 2015 surveys, and HIV-seronegative participants aged 15 to 35 years were followed up in the 2016 and 2017 cohorts. Analysis was conducted from January 1 through December 31, 2018.

**Exposures:**

HIV prevention and treatment programs in a real-world, nontrial setting.

**Main Outcomes and Measures:**

Trends in sex- and age-specific HIV incidence rates, condom use, voluntary medical male circumcision, knowledge of HIV-seropositive status, uptake of antiretroviral therapy, and viral suppression.

**Results:**

A total of 9812 participants (6265 women [63.9%]; median age, 27 years [interquartile range, 20-36 years]) from 11 289 households were enrolled in the 2014 survey, and 10 236 participants (6341 women [61.9%]; median age, 27 years [interquartile range, 20-36 years]) from 12 247 households were enrolled in the 2015 survey. Of these, 3536 of 4539 (annual retention rate of 86.7%) completed follow-up in the 2016 cohort, and 3907 of 5307 (annual retention rate of 81.4%) completed follow-up in the 2017 cohort. From 2014 to 2015, condom use with last sex partner decreased by 10% from 24.0% (n = 644 of 3547) to 21.6% (n = 728 of 3895; *P* = .12) in men and by 17% from 19.6% (n = 1039 of 6265) to 16.2% (n = 871 of 6341; *P* = .002) in women. Voluntary medical male circumcision increased by 13% from 31.9% (1102 of 3547) to 36.1% (n = 1472 of 3895); *P* = .007) in men, and the proportion of women reporting that their partner was circumcised increased by 35% from 35.7% (n = 1695 of 4766) to 48.2% (n = 2519 of 5207; *P* < .001). Knowledge of HIV-seropositive status increased by 21% from 51.8% (n = 504 of 3547) to 62.9% (n = 570 of 3895; *P* < .001) in men and by 14% from 64.6% (n = 1833 of 6265) to 73.4% (n = 2182 of 6341; *P* < .001) in women. Use of antiretroviral therapy increased by 32% from 36.7% (n = 341 of 3547) to 48.6% (n = 432 of 3895; *P* < .001) in men and by 29% from 45.6% (n = 1251 of 6265) to 58.8% (n = 1743 of 6341; *P* < .001) in women; HIV viral suppression increased by 20% from 41.9% (n = 401 of 3547) to 50.3% (n = 456 of 3895; *P* = .005) in men and by 13% from 54.8% (n = 1547 of 6265) to 61.9% (n = 1828 of 6341; *P* < .001) in women. Incidence of HIV declined in women aged 15 to 19 years from 4.63 (95% CI, 3.29-6.52) to 2.74 (95% CI, 1.84-4.09) per 100 person-years (*P* = .04) but declined marginally or remained unchanged among men and women in other age groups.

**Conclusions and Relevance:**

This study showed a significant decline in HIV incidence in young women; however, to further reduce HIV incidence, HIV prevention and treatment program coverage must be intensified and scaled up.

## Key Points

**Question:**

What are the trends in the coverage of HIV prevention and treatment programs and HIV incidence in a hyperendemic area of KwaZulu-Natal, South Africa?

**Findings:**

This community-based cohort study of 2 sequential surveys in 9812 and 10 236 respondents showed that HIV incidence in young women (aged 15-19 years) declined significantly from 4.63 to 2.74 per 100 person-years, but declines were marginal or remained unchanged among men and women in other age groups. In parallel, the uptake of medical male circumcision, knowledge of HIV-positive status, antiretroviral therapy, and viral suppression increased.

**Meaning:**

These findings suggest that, to further reduce HIV incidence, prevention and treatment program coverage must be intensified and scaled up.

## Teaser

This community-based cohort study examines trends in coverage of HIV prevention and treatment programs and HIV incidence among adults in the hyperendemic area of KwaZulu-Natal, South Africa.

## Introduction

In Africa, ongoing high HIV incidence in young women^1,2,3^ is the leading obstacle to achieving the United Nations goal of global epidemic control by the year 2030.^4^ In South Africa, the world’s worst-affected country, approximately 7.9 million people of all ages were living with HIV in 2017,^5^ and among adults aged 15 to 49 years, HIV prevalence was 20.6% (26.3% among women and 14.8% among men). KwaZulu-Natal is the worst-affected province, with a prevalence of 27.0% compared with the western Cape, which has a prevalence of 12.6%. Several studies from KwaZulu-Natal have shown persistently high HIV prevalence^6,7,8,9^ and incidence^10^ in young women, suggesting that the burden of HIV continues unabated.^3,8,11^

In 2010, the South African Department of Health progressively scaled up HIV prevention and treatment programs. These programs included access to HIV testing services with linkage to care, prevention of mother-to-child transmission of HIV,^12^ voluntary medical male circumcision (VMMC),^13^ provision of HIV preexposure and postexposure prophylaxis,^14,15,16^ antiretroviral therapy (ART), and a treatment-as-prevention^15^ strategy to improve HIV-related morbidity and mortality, increase life expectancy,^17,18^ and reduce HIV transmission potential.^17,19,20^ To accelerate the response toward achieving the goal to HIV epidemic control and to finally end the AIDS epidemic, the Joint United Nations Programme on HIV/AIDS (UNAIDS) 90-90-90 treatment target measures (ie, require 90% of all people living with HIV to know their HIV status, 90% of all people with diagnosed HIV infection to receive sustained ART, and 90% of all people receiving ART to achieve viral suppression),^21,22^ universal test-and-treat strategy,^23^ and HIV self-testing strategy^24^ have been implemented. Furthermore, criteria for initiation of ART, ART regimens, use of mobile clinics, nurse-initiated management of ART, and use of a fixed-dose drug combination have been implemented to streamline treatment, improve adherence, and achieve and sustain viral suppression.^15^

Despite this scale-up, these programs to date^5^ have had little effect on HIV incidence and the cycle of HIV transmission^25^ created by young women’s age-disparate sexual partnerships in KwaZulu-Natal.^25,26^ The objectives of this study were to assess the trends in the coverage of HIV prevention and treatment programs and HIV incidence in a hyperendemic HIV epidemic setting in KwaZulu-Natal, South Africa.

## Methods

### Study Setting and Design

The HIV Incidence Provincial Surveillance System was a platform designed to measure HIV prevalence and incidence in association with the scale-up of prevention and treatment efforts in a real-world, nontrial setting in rural Vulindlela and periurban Greater Edendale areas in the uMgungundlovu district of KwaZulu-Natal, South Africa.^27^ This cohort study was approved by the Biomedical Research Ethics Committee of the University of KwaZulu-Natal, the KwaZulu-Natal Provincial Department of Health, and the Centers for Disease Control and Prevention, Atlanta, Georgia. All enrolled participants provided written informed consent. The study followed the Strengthening the Reporting of Observational Studies in Epidemiology (STROBE) reporting guidelines.^28^

The study communities have a population of approximately 360 000, are predominantly Zulu speaking, and are characterized by high levels of unemployment, poverty, and teenage pregnancy and high rates of HIV.^6,7,8^ Health care is provided through nurse-run, public-sector primary health care clinics, district hospitals, and community-based organizations. External agencies, including the US President’s Emergency Plan for AIDS Relief, fund district partners to support the HIV prevention and treatment program implementation activities and provide technical support to strengthen health services. Although women routinely access HIV services when attending local clinics for reproductive health care, men seldom use these services^29^ and are a difficult-to-reach group for provision of universal test-and-treat strategies.^30^

The KwaZulu-Natal Department of Health coordinates its community-related HIV services and programs through the KwaZulu-Natal government’s Operation *Sukuma Sakhe* (Zulu for *stand up and build*)^31,32^ and established key partnerships with stakeholders to implement outreach campaigns, including the *Isibaya samaDoda* campaign, meaning *including/bringing in the men* in Zulu.^33^ These campaigns were initiated to enhance cooperation and facilitate scale-up of HIV prevention programs and strengthen services to reach and enhance HIV health care. Furthermore, the campaigns focused on information and education on improving sexual and reproductive health, knowledge of HIV status, access to HIV prevention and treatment programs, and on helping create, support, and sustain demand for VMMC for all men regardless of age.^34^

### Study Sampling and Procedures

Two sequential, community-based household surveys were undertaken from June 11, 2014, to June 22, 2015 (2014 survey), and from July 8, 2015, to June 7, 2016 (2015 survey). Age-eligible, HIV-seronegative participants from the 2014 and 2015 surveys had a single follow-up visit from June 24, 2016, to April 3, 2017 (2016 cohort), or from November 7, 2016, to August 30, 2017 (2017 cohort), respectively. The sequential surveys measured HIV prevalence and assessed exposure to HIV prevention and treatment programs, whereas the sequential, prospective cohorts measured HIV incidence rates.^27^

We used a multistage cluster sampling method to randomly select census enumeration areas. Within each census enumerator area, households were randomly selected, and a single age-eligible (15-49 years) individual per consenting household was selected for study participation. The sample selection procedures have been published previously.^27^ For each consenting individual, a structured questionnaire was administered to collect sociodemographic data, psychosocial data, sexual behavior, male circumcision status, HIV testing history, and exposure to districtwide, public-sector HIV prevention and treatment programs. Peripheral blood samples were collected for laboratory measurements and storage. Global positioning system coordinates and fingerprint biometrics were used to facilitate finding of homes and confirming the identity of eligible participants for the follow-up visit. Participants were considered lost to follow-up after 3 unsuccessful contact attempts. Individuals aged 36 to 49 years who were HIV seronegative were not included in the cohorts because of the expected low HIV incidence rates in this age group.^27^

We measured HIV antibodies using the fourth-generation HIV enzyme-linked immunosorbent assay (Vironostika Uniform II Antigen/Antibody MicroELISA system; bioMérieuxe), and positive findings were confirmed with the HIV-1/HIV-2 assay (Combi Elecys; Roche Diagnostics) and an HIV-1 Western blot assay (Bio-Rad Laboratories). HIV-1 RNA viral load (dynamic range, 20 copies/mL to 10 million copies/mL) was measured using a nucleic acid amplification test for HIV-1 (COBAS AmpliPrep/COBAS TaqMan HIV-1 assay, version 2.0; Roche Diagnostics). Participants with HIV-seropositive results at the follow-up visit had their positive and baseline stored samples tested simultaneously for viral load and antibodies to confirm HIV seroconversion. Use of antiretroviral (ARV) drugs lamivudine, emtricitabine, nevirapine, efavirenz, and lopinavir was measured by mass spectrometry using electrospray ionization-positive mode (QTRAP 6500+; AB SCIEX) in the plasma of a sample of participants (n = 343) to assess the accuracy of self-reported ARV drug use.

HIV-1 polymerase sequences from samples with viral load greater than 1000 copies/mL from both surveys and both cohorts were subtyped using Genome Detective.^35^ All subtype C sequences (n = 3123) were aligned and manually edited after the removal of 33 codon positions associated with drug resistance. The subsequent alignment was then used to identify the best-fitting nucleotide substitution model (GRT + G + I) in the jModelTest.^36^ Phylogenetic reconstruction^37^ and cluster identification^38^ were performed as previously described.^25^

### Statistical Analysis

Data were analyzed from January 1, 2018, through December 31, 2018. Statistics were weighted to account for the complex sampling design and adjusted for nonresponse in the surveys.^8,39^ Standard errors of estimates were estimated using Taylor series linearization methods, from which Wald 95% confidence limits were derived. Changes in coverage in self-reported condom use and VMMC among all participants and changes in knowledge of HIV-seropositive status, self-reported ART use, and viral suppression among all laboratory-diagnosed, HIV-seropositive participants were estimated and assessed using 2-sided, unpaired *t* tests. Viral suppression was defined as HIV RNA of less than 400 copies/mL. Level of ARV drugs was measured in the plasma of a sample of participants to assess the accuracy of self-reported ARV drug use.

Measurement of HIV incidence rate accounted for the duration of risk, defined as the time from the date of enrollment to the date of exit from the cohort. For HIV seroconversions, the estimated date of infection was the midpoint between the last HIV-seronegative test finding and the first HIV-seropositive test finding. Using age at enrollment in each of the surveys, sex- and age-stratified analyses were undertaken for HIV incidence rates. Incidence rate ratios (IRRs) have been adjusted for potential confounders, including sex, age, educational level, number of lifetime sex partners, and HIV testing history. The adjusted IRRs (aIRRs) were calculated using Poisson regression models performed in Stata, version 13 (StataCorp LLC). Incidence rates during the follow-up period may not be assumed to be constant^40^; therefore, a sensitivity analysis was conducted to adjust for the longer follow-up time in the 2016 cohort. Approximately 10% (n = 301) of participants from the 2016 cohort (those with longer follow-up times than the maximum follow-up time observed in the 2017 cohort) were excluded, ensuring that the distribution of follow-up time was comparable across the cohorts. All statistical analyses were conducted in SAS, version 9.4 (SAS Institute Inc) unless otherwise stated. Two-sided *P* < .05 indicated statistical significance.

## Results

**Figure 1.**
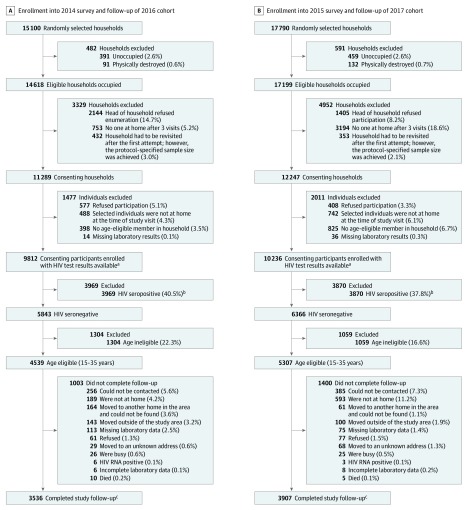
Recruitment, Enrollment, and Follow-up of Study Participants Percentages are unweighted. ^a^HIV prevention efforts (eg, self-reported condom use with last sex partner, medical male circumcision, and knowledge of HIV-seropositive status) were assessed in these individuals. ^b^Antiretroviral therapy coverage and viral suppression were assessed in HIV-seropositive individuals. ^c^HIV seroconversions were assessed in these individuals.

Figure 1 shows the recruitment and enrollment of study participants in the 2014 and 2015 surveys and of participants followed up in the 2016 and 2017 cohorts. Of the 15 100 households randomly selected for the 2014 survey, 11 289 consented, and 9812 participants (response rate of 86.9%) were enrolled (6265 women [63.9%] and 3547 men [36.1%]; median age, 27 years [interquartile range, 20-36 years]). Of the 17 790 households randomly selected for the 2015 survey, 12 247 participants consented, and 10 236 (response rate of 83.6%) were enrolled (6341 women [61.9%] and 3895 men [38.1%]; median age, 27 years [interquartile range, 20-36 years]). Among the HIV-seronegative, age-eligible participants from the 2014 survey, 3536 of 4539 (annual retention rate of 86.7%) were followed up for a mean of 20 months (range, 12-33 months). Similarly, of the 5307 HIV-seronegative, age-eligible participants from the 2015 survey, 3907 (annual retention rate of 81.4%) were followed up for a mean of 17 months (range, 9-26 months). Two hundred ninety individuals (3.0% for the 2014 survey and 2.8% for the 2015 survey) were independently included in both surveys, 113 of whom were in both cohorts; excluding them from the analysis did not change the outcome.

Table 1 shows the characteristics of all participants in the 2014 and 2015 surveys and those of HIV-seronegative, age-eligible participants followed up in the 2016 and 2017 cohorts. Across both surveys, a higher percentage of women were enrolled. Median ages of men and women were comparable across the surveys and across the cohorts; however, the median age of individuals enrolled in the cohorts was younger than that of the individuals included in the surveys. Educational levels and rates of marriage in the community were low. Weighted prevalence of HIV was 36.3% (n = 3969) in the 2014 survey and 35.2% (n = 3870) in the 2015 survey. More than one-third of HIV-seropositive men and more than one-fifth of HIV-seropositive women were found to have CD4 cell counts of less than 350/μL. In addition, among HIV-seropositive men, 143 (23.4%) in the 2014 survey and 218 (14.8%) in the 2015 survey had a viral load of at least 100 000 copies/mL; among HIV-seropositive women, 154 (33.3%) in the 2014 survey and 262 (23.6%) in the 2015 survey had a viral load of at least 100 000 copies/mL.

**Table 1.** Baseline Characteristics of Participants in the 2014 and 2015 Surveys and in the 2016 and 2017 Cohorts^a^

Characteristic	Enrolled Participants				Enrolled HIV-Seronegative, Age-Eligible Participants			
	2014 Survey (n = 9812)		2015 Survey (n = 10 236)		2016 Cohort (n = 3536)		2017 Cohort (n = 3907)	
	Men (n = 3547)	Women (n = 6265)	Men (n = 3895)	Women (n = 6341)	Men (n = 1573)	Women (n = 1963)	Men (n = 1829)	Women (n = 2078)
**Sociodemographic**								
Age, median (IQR), y	26.4 (20.1-35.0)	27.4 (20.6-36.2)	26.4 (20.1-34.9)	27.3 (20.7-36.2)	21.4 (18.2-26.4)	20.8 (17.7-25.8)	21.9 (18-26.5)	21.1 (17.5-25.3)
Age distribution, y								
15-19	658 (19.6)	958 (18.2)	875 (19.6)	1076 (18.2)	518 (31.5)	660 (36.7)	668 (30.2)	743 (36.2)
20-24	814 (20.8)	1266 (19.5)	901 (20.8)	1255 (19.5)	537 (31.5)	639 (30.8)	572 (30.5)	668 (32.4)
25-29	602 (18.2)	1087 (17.9)	638 (18.2)	1167 (17.9)	306 (21.8)	419 (20.0)	339 (23.6)	416 (20.2)
30-34	461 (13.9)	833 (13.7)	540 (13.9)	972 (13.7)	212 (15.2)^b^	245 (12.5)^b^	250 (15.7)^b^	251 (11.3)^b^
35-39	405 (12.3)	760 (12.3)	379 (12.3)	729 (12.3)	NA	NA	NA	NA
40-44	320 (8.6)	660 (9.6)	317 (8.6)	634 (9.6)	NA	NA	NA	NA
45-49	287 (6.5)	701 (8.9)	245 (6.5)	508 (8.9)	NA	NA	NA	NA
Completed secondary school	1613 (43.5)	2948 (46.6)	1723 (44.5)	2933 (45.9)	783 (48.8)	1083 (53.1)	807 (46.4)	1057 (51.2)
Married	180 (5.9)	682 (11.7)	251 (8.3)	855 (13.3)	12 (1.2)	106 (6.0)	19 (1.2)	107 (4.7)
**Behavioral**								
Ever had sex	2855 (80.8)	5447 (85.6)	3275 (85.0)	716 (87.6)	1122 (72.0)	1463 (72.1)	1361 (75.1)	1571 (73.4)
Age at sexual debut, median (IQR), y	17 (16-18)	18 (17-20)	17 (15-18)	18 (16-19)	17 (16-18)	18 (17-19)	17 (15-18)	18 (16-19)
Age of partner at sexual debut, median (IQR), y	17 (15-18)	21 (19-24)	16 (15-18)	21 (19-24)	16 (15-16)	19 (18-22)	16 (15-18)	20 (18-23)
≥2 Lifetime sex partners^c^	1955 (65.1)	3133 (55.6)	2830 (74.3)	4115 (64.0)	781 (56.9)	728 (38.0)	1148 (65.8)	949 (44.9)
Currently in a sexual relationship	2459 (68.6)	4736 (74.1)	2792 (73.3)	4938 (76.4)	952 (62.0)	1321 (64.8)	1139 (64.5)	1414 (66.7)
Currently in a relationship with ≥2 partners	206 (7.5)	63 (1.1)	457 (12.2)	111 (1.7)	107 (8.6)	18 (0.85)	204 (12.2)	40 (1.8)
Always used condoms with last sex partner^d^	644 (24.0)	1039 (19.6)	728 (21.6)	871 (16.2)	310 (29.8)	281 (18.8)	360 (25.9)	263 (18.0)
**Clinical**								
Medically circumcised	1102 (31.9)	NA	1472 (36.1)	NA	697 (43.8)	NA	885 (46.2)	NA
Ever pregnant	NA	4391 (70.7)	NA	4738 (73.5)	NA	1143 (55.4)	NA	1211 (56.2)
Currently pregnant as proportion of women aged 15-35 y^e^	NA	303 (6.8)	NA	292 (6.2)	NA	144 (6.5)	NA	135 (6.3)
Ever told by a physician that you have STI(s)	231 (7.7)	318 (6.2)	336 (8.7)	523 (8.2)	85 (6.3)	67 (4.1)	138 (7.8)	121 (5.4)
Ever told by a physician that you have tuberculosis	203 (6.5)	274 (4.5)	262 (7.3)	422 (6.5)	21 (1.7)	14 (0.6)	30 (1.6)	35 (1.3)
HIV testing history								
Ever tested	2326 (68.8)	4939 (81.9)	3193 (81.9)	5854 (91.4)	1027 (67.1)	1473 (76.0)	1439 (78.9)	1790 (85.2)
HIV seropositive^e^	1014 (28.0)	2955 (44.1)	922 (24.5)	2948 (45.0)				
Knows HIV-seropositive status	504 (51.8)	1833 (64.6)	570 (62.9)	2182 (73.4)				
CD4 cell count, No./μL^e^^,^^f^								
<350	439 (41.0)	696 (23.1)	333 (34.5)	634 (21.6)	NA	NA	NA	NA
350-499	243 (23.9)	639 (21.1)	241 (27.1)	576 (19.7)	NA	NA	NA	NA
≥500	327 (35.1)	1593 (55.8)	343 (38.4)	1729 (58.6)	NA	NA	NA	NA
Using ART, No. (weighted %) of HIV-seropositive participants	341 (36.7)	1251 (45.6)	432 (48.6)	1743 (58.8)	NA	NA	NA	NA
Viral suppression, No. (weighted %) of HIV-seropositive participants^e^^,^^g^	401 (41.9)	1574 (54.8)	456 (50.3)	1828 (61.9)	NA	NA	NA	NA
HIV viral load distribution, No. (weighted %) of HIV-seropositive participants^e^^,^^g^								
400-1000	26 (3.8)	23 (4.6)	106 (8.1)	102 (9.6)	NA	NA	NA	NA
>1000-10 000	131 (18.6)	110 (23.5)	408 (29.1)	333 (29.3)	NA	NA	NA	NA
>10 000-100 000	309 (54.2)	178 (38.6)	640 (48.0)	422 (37.5)	NA	NA	NA	NA
>100 000	143 (23.4)	154 (33.3)	218 (14.8)	262 (23.6)	NA	NA	NA	NA

Abbreviations: ART, antiretrovial therapy; IQR, interquartile range; NA, not applicable; STI, sexually transmitted infection.

^a^ Unless otherwise indicated, data are expressed as number (weighted percentages), to account for the multilevel sampling design.

^b^ Includes those aged 35 years. Individuals aged 36 to 49 years who were HIV seronegative were not included in the cohorts because of the expected low HIV incidence rates in this age group.

^c^ Percentages exclude those who refused to report their lifetime number of sexual partners (refusal rates were 13% and <0.5% in the 2014 and 2015 surveys, respectively).

^d^ Based on the percentage of all individuals reporting to be sexually active.

^e^ Based on laboratory measurement.

^f^ A total of 5 men and 27 women in the 2014 survey and 5 men and 9 women in the 2015 survey were missing CD4 cell count data and are excluded.

^g^ Defined as HIV-1 RNA viral load of less than 400 copies/mL. A total of 4 men and 9 women in the 2014 survey and 1 man and 1 woman in the 2015 survey were missing viral load data and are excluded.

**Figure 2.**
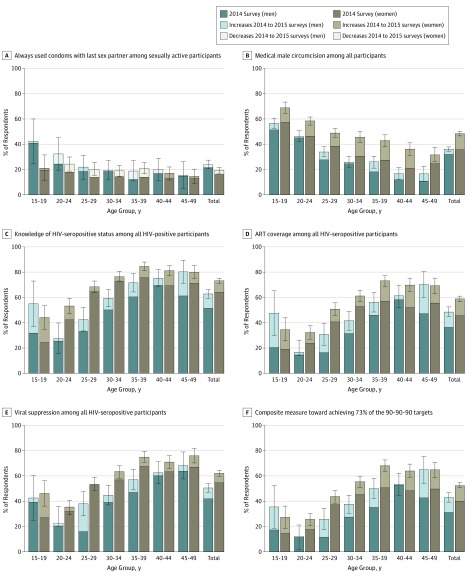
Panel Showing Trends in Community Coverage of Selected HIV Prevention and Treatment Programs, Totals and by Sex and Age Group Data are given as totals and stratified by sex and age group. Error bars indicate 95% CI. A, Use of condoms is self-reported by men who report condom use and women who report their partner using condoms. B, Men reported being medically circumcised and women reporting their partner being medically circumcised. C and D, Knowledge of HIV-seropositive status and use of antiretroviral therapy (ART) were self-reported among all participants with laboratory-confirmed HIV-seropositive status. E, Viral suppression was defined as HIV RNA level of less than 400 copies/mL among all participants with laboratory-confirmed HIV-seropositive status. F, Composite measure of the Joint United Nations Programme on HIV/AIDS (UNAIDS) 90-90-90 targets of diagnosis, treatment coverage, and viral suppression indicates that 90% of all HIV-seropositive people are diagnosed, 90% of those diagnosed receive treatment (81%), and 90% of those treated have achieved viral suppression (73%), resulting in 73% of all HIV-seropositive people receiving ART achieving viral suppression.

Figure 2 and eTable 1 in the Supplement show the considerable variability by sex and age group in the trends in community coverage of selected HIV prevention and treatment programs in the 2014 to the 2015 surveys. The number of participants who reported always having used condoms with their last sex partner decreased by 10% from 644 (24.0%) to 728 (21.6%; *P* = .12) in men and by 17% from 1039 (19.6%) to 871 (16.2%; *P* = .002) in women. Voluntary male medical circumcisions increased by 13% from 1102 (31.9%) to 1472 (36.1%; *P* = .007) in men and by 35% from 1695 (35.7%) to 2519 (48.2%; *P* < .001) in women who reported that their partner was circumcised. The number of individuals reporting knowledge of HIV-seropositive status increased by 21% from 504 (51.8%) to 570 (62.9%; *P* < .001) in men and by 14% from 1833 (64.6%) to 2182 (73.4%; *P* < .001) in women. The number of individuals self-reporting ART use increased by 32% from 341 (36.7%) to 432 (48.6%; *P* < .001) in men and by 29% from 1251 (45.6%) to 1743 (58.8%; (*P* < .001) in women. The self-reported ART data were considered a good proxy for ART use because, from the sample of 343 participants selected for ARV testing, ARV drugs were detected in 64 of 71 (90.1%) of those reporting use of ARV drugs and in 34 of 272 (12.5%) of those not reporting use of ARV drugs. From the 2014 to the 2015 surveys, cases of HIV viral suppression also increased by 20% from 401 (41.9%) to 456 (50.3%; *P* = .005) in men and by 13% from 1547 (54.8%) to 1828 (61.9%; *P* < .001) in women. Assessment of the UNAIDS 90-90-90 targets composite measure showed that this measure increased by 38% from 284 (30.8%) to 376 (42.6%; *P* < .001) in men and by 32% from 1086 (39.9%) to 1545 (52.5%; *P* < .001) in women.

Table 2 presents the HIV seroconversions and incidence rates by sex and age group. In young women (aged 15-19 years), the HIV incidence rate declined from 4.63 (95% CI, 3.29-6.52) per 100 person-years to 2.74 (95% CI, 1.84-4.09) per 100 person-years, a decline of 43% from the 2016 to the 2017 cohorts (aIRR, 0.57; 95% CI, 0.34-0.96; *P* = .04). However, the HIV incidence rates declined marginally or remained unchanged among men and women in other age groups. The overall HIV incidence rate was 2.31 (95% CI, 1.82-2.92) per 100 person-years in the 2016 cohort and declined to 1.96 (95% CI, 1.62-2.37) per 100 person-years in the 2017 cohort (aIRR, 0.86; 95% CI, 0.63-1.18; *P* = .35). The overall changes in HIV incidence rates in men (aIRR, 0.95; 95% CI, 0.53-1.70; *P* = .85) and women (aIRR, 0.82; 95% CI, 0.58-1.16; *P* = .26) were minimal and not statistically significant. Sensitivity analysis adjusting for differences in the follow-up times in the 2016 and 2017 cohorts did not alter findings (eTable 2 in the Supplement).

**Table 2.** Trends in HIV Seroconversions^a^

Age Group, y	2016 Cohort			2017 Cohort			Relative Change in HIV Incidence			
	HIV Seroconversions, No./Total No.	No. of Person-Years	IR per 100 Person-Years (95% CI)^b^	HIV Seroconversions, No./Total No.	No. of Person-Years	IR per 100 Person-Years (95% CI)^b^	IRR (95% CI)	*P* Value	Adjusted IRR (95% CI)^c^	*P* Value
All	163/3536	5746	2.31 (1.82-2.92)	115/3907	5447	1.96 (1.62-2.37)	0.85 (0.63-1.15)	.28	0.86 (0.63-1.18)	.35
**Men**										
All	39/1573	2602	1.44 (0.96-2.24)	31/1829	2560	1.32 (0.90-1.92)	0.92 (0.51-1.65)	.78	0.95 (0.53-1.70)	.85
15-19	4/518	836	0.41 (0.14-1.21)	3/668	940	0.24 (0.08-0.75)	0.59 (0.12-2.79)	.50	0.58 (0.12-2.82)	.50
20-24	16/537	888	1.42 (0.76-2.65)	11/572	800	1.18 (0.61-2.27)	0.83 (0.34-2.06)	.69	0.88 (0.35-2.22)	.79
25-29	13/306	523	2.46 (1.18-5.15)	13/339	467	2.84 (1.57-5.14)	1.15 (0.45-2.97)	.77	1.23 (0.46-3.31)	.68
30-35	6/212	355	2.02 (0.69-5.96)	4/250	354	1.45 (0.52-4.03)	0.72 (0.16-3.18)	.66	0.72 (0.16-3.16)	.66
**Women**										
All	124/1963	3144	3.44 (2.71-4.38)	84/2078	2887	2.80 (2.22-3.53)	0.81 (0.58-1.13)	.22	0.82 (0.58-1.16)	.26
15-19	51/660	1039	4.63 (3.29-6.52)	30/743	1032	2.74 (1.84-4.09)	0.59 (0.35-1.00)	.05	0.57 (0.34-0.96)	.04
20-24	41/639	1022	4.00 (2.74-5.85)	41/668	923	4.26 (3.04-5.97)	1.07 (0.64-1.77)	.81	1.07 (0.64-1.80)	.79
25-29	26/419	680	2.29 (1.43-3.67)	10/416	580	1.87 (0.93-3.77)	0.82 (0.35-1.90)	.64	0.85 (0.36-1.97)	.70
30-35	6/245	403	0.59 (0.26-1.37)	3/251	352	0.47 (0.15-1.50)	0.80 (0.19-3.32)	.75	0.85 (0.20-3.60)	.83

Abbreviations: IR, incidence rate; IRR, IR ratio.

^a^ A total of 278 Western blot analyses confirmed HIV seroconversions: 163 in the 2016 cohort and 115 in the 2017 cohort. This total excluded the 1 Western blot–unconfirmed positive result of enzyme-linked immunosorbent assay (ELISA) and 9 ELISA seroconversions among participants who were found to be HIV RNA positive at enrollment.

^b^ Weighted to account for the multilevel sampling design, estimated using survey Poisson regression models, and reported as per 100 person-years.

^c^ Adjusted for sex, age, educational level, and lifetime number of sex partners plus HIV testing history.

**Figure 3.**
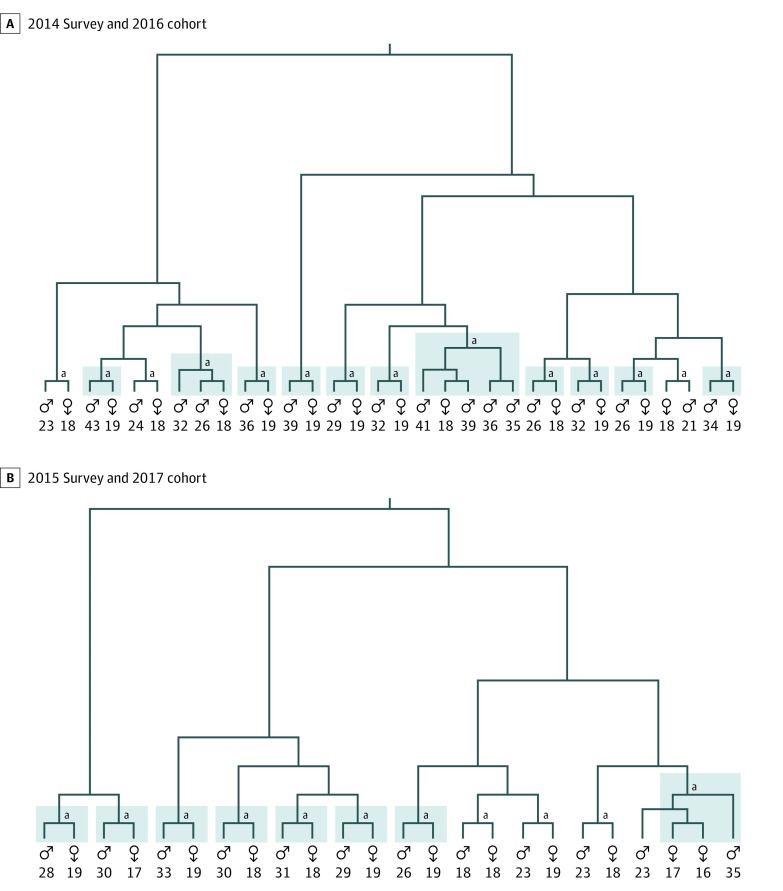
Maximum Likelihood Tree for Heterosexual Transmission Clusters Involving at Least 1 Young Woman Aged 15 to 19 Years Linkages with a man 25 years or older are highlighted in blue. For better visualization of the clusters, the tree is represented with proportional branch length transformation. The sex and age (in years) of individuals in a transmission cluster are included. Clusters were identified with support higher than 90% and genetic diversity lower than 4.5% from a data set of 3123 sequences. ^a^Root of the cluster.

Figure 3 shows the maximum likelihood tree for heterosexual transmission clusters. Of the HIV-1 viral sequences, 1655 sequences from the 2014 survey and 107 sequences from the 2016 cohort generated 163 heterosexual clusters (includes ≥1 man and ≥1 woman). Eighteen of these linked clusters involved young women, and the mean age of men in these clusters was 31.9 years. Similarly, 1283 sequences from the 2015 survey and 78 from the 2017 cohort generated 160 heterosexual clusters. Thirteen of these linked clusters involved young women, and the mean age of men in these clusters was 27.1 years. The mean age difference between young women and linked men declined from 13.5 years in the 2014 survey and 2016 cohort to 9.3 years in the 2015 survey and 2017 cohort (*P* = .046). Furthermore, young women were linked to a man 25 years or older in 15 of 18 clusters (83.3%) in the 2014 survey and 2016 cohort and 8 of 13 clusters (61.5%) in the 2015 survey and 2017 cohort (*P* = .23).

## Discussion

Preventing new HIV infections in young women has been one of the greatest challenges in Africa,^10,22,41,42^ with little success to date.^43^ Implementation of programs based on knowledge of the local epidemic^44^ is key to accessing hard-to-reach groups of individuals and, in particular, to reaching older men, who play an important role in underlying HIV transmission dynamics.^25^ Reaching men and treating them for HIV infection in these settings has not been easy, as seen by the challenges in achieving high treatment coverage among men in a large test-and-treat trial in another KwaZulu-Natal community.^45^ This community-based longitudinal study provides empirical evidence of a decline in HIV incidence among young women (aged 15-19 years). The observed decline in HIV incidence in young women was unlikely to be due to HIV preexposure prophylaxis because preexposure prophylaxis was not available through government clinics during the study period.^16^ The decline could not be due to changes in risk behavior because age of sexual debut, number of lifetime sex partners, and condom use remained relatively unchanged. An analysis of phylogenetic clustering of HIV sequences showed that the mean age difference of young women linked to men in age-disparate sexual partnerships declined from 13.5 to 9.3 years. These findings suggest that, although risk behavior was unlikely to have changed, increases in the number of older HIV-seropositive men in age-disparate sexual partnerships who were using ART and had viral suppression may have led to the reduction observed in HIV incidence.

Although the decrease in HIV incidence among young women to 2.74 per 100 person-years is reassuring, it is far from what is needed for epidemic control and from the HIV elimination target of 1.00 per 1000 person-years.^46^ Moreover, overall incidence rates in the area failed to decline. The need is overwhelming for an intensified scale-up of programs in this area.

In this study, we observed an increase of 13% in VMMC from 31.9% to 36.1% in men and an increase of 35% in the number of women reporting that their most recent partner was circumcised. However, minimal declines in HIV incidence were observed among men. The benefits of VMMC are well established. Increasing VMMC has the potential to substantially reduce risk of HIV acquisition in men, and modeling data suggest that women benefit indirectly from reduced HIV prevalence in circumcised male partners.^47^ Studies from Uganda^48^ and Kenya^49^ have shown the protective effect of VMMC among young boys and men. With VMMC coverage of more than 70% in western Kenya, the protective effect of MMC was shown for prevalent infections, although this effect was not significant for incident infections.^49^ Importantly, women and girls reporting a circumcised partner were shown to be less likely to acquire HIV. In Uganda in communities with a median coverage of 39%, VMMC was associated with a lower HIV incidence in men.^48^ Collectively, the findings from these studies show that maximizing the rapid scale-up of VMMC benefits men and, in the longer term, women^47,48,49,50^; therefore, using mathematical modeling to inform health policy, a framework for successful scale-up of VMMC has been proposed.^51^

Treatment for HIV has been a priority in South Africa, and as the UNAIDS 90-90-90 targets gain momentum, the role of HIV testing services has never been more important. Testing for HIV significantly improved knowledge of HIV-seropositive status during the observation period. However, knowledge of HIV status was still low in young men and women, and many of these individuals have the potential to sustain the epidemic. Although significant improvement in the uptake of ART occurred, only 48.6% of men and 58.8% of women self-reported use of ART in the 2015 survey. In addition, uptake of ART varied considerably with age, with a lower proportion of younger men and women reporting its use and thereby highlighting the gaps in the reach of ART. Given the efficacy of ART in reducing and suppressing HIV viral load, it appears that ART scale-up is imperative to reducing HIV transmission rates in this area.

Viral suppression of HIV among all HIV-seropositive participants was 50.3% in men and 61.9% in women in 2015. In contrast, using the same threshold of HIV RNA of less than 400 copies/mL, the UNAIDS criteria to assess progress in controlling the HIV epidemic showed that viral suppression was achieved through ART in 42.6% of men and 52.5% of women. Differences in these measures may be attributable to failure of participants to report their HIV-seropositive status or ART use to study staff for fear of being stigmatized or experiencing discrimination.^46^ However, we found that, among those who self-reported ART use, detectable levels of ARV drugs could be found in 90.1%. The HIV viral load is an important factor associated with HIV transmission,^19^ and increasing viral suppression in the community therefore has the potential to interrupt onward transmission.^17,20^ Several studies^17,19,20,52^ provide compelling evidence that viral suppression or lowered viral load reduces the potential of HIV transmission to sexual partners. However, making a difference in viral suppression through generalized, community-based test-and-treat interventions is challenging if groups of individuals who are key to HIV transmission in a community are not adequately reached.^53^ Individuals with very high viral loads, most likely owing to acute or early HIV infection, may not be readily identifiable through test-and-treat strategies.^53^ Recently completed population-based trials have provided mixed results. The trial of an HIV test-and-treat intervention in Uganda and Kenya found no reduction in HIV incidence despite an 11% (68% vs 79%) difference in viral suppression.^54^ However, in Botswana, a 31% lower HIV incidence rate was found in intervention communities, where viral suppression increased from 70% to 88% compared with control communities, where it increased from 75% to 83%.^55^ In Zambia and South Africa, the HPTN 071 cluster-randomized trial^56^ reported a 30% reduction in HIV incidence from implementing prevailing HIV treatment guidelines with community outreach. Surprisingly, the same trial showed no effect on HIV incidence from a universal test-and-treat intervention with community outreach.^56^ In the present study, even with a 20% and 13% increase in viral suppression among men and women, respectively, we observed no overall decline in HIV incidence because the proportion of men with extremely high viral load of more than 100 000 copies/mL remained high. Therefore, a targeted approach to maximize coverage of HIV prevention and treatment programs to affect HIV incidence rates is needed.

### Strengths and Limitations

A key strength of the study was that the cohorts were drawn from separate periods from independent representative community samples, and therefore the observed increase in the uptake of HIV prevention and treatment programs reflects the real-world implementation efforts to increase coverage. The robustness of the study design, high participation and retention rates, biological laboratory measurements, and HIV incidence measurement based on HIV seroconversions underscore the high quality of our study. The sex-age disaggregated data analyses were important to identify gaps in the programs and groups of individuals not accessing programs.

Our results are limited to the study areas and not necessarily generalizable beyond communities with substantial epidemics among heterosexual individuals, although our findings may be applicable to many parts of the African continent where age-disparate sexual transmissions are common and coverage of programs is limited.^3,26,57,58^ Our results cannot be used to draw conclusions about the sustainability of the effects of outreach campaigns, highlighting the need for robust ongoing surveillance to assess the long-term positive trends observed in this study.

## Conclusions

Findings from this study are encouraging. The trends in the coverage of HIV prevention and treatment programs in a real-world, nontrial setting supported through community outreach campaigns showed significant increases in VMMC, knowledge of HIV-seropositive status, uptake of ART, and viral suppression and, importantly, a decline in HIV incidence in young women. However, our results also suggest an urgent need to intensify the scale-up of programs, including preexposure prophylaxis, to further reduce HIV incidence.

## Notes

Supplement. **eTable 1.** Trends in Community Coverage of Selected HIV Prevention and Treatment Programs Overall and by Sex and Age Group **eTable 2.** Sensitivity Analysis Adjusting for Differences in the Follow-up Times of Participants in the 2016 and 2017 Cohorts
