# Supplementary material for: Trends in HIV Prevention, Treatment, and Incidence in a Hyperendemic Area of KwaZulu-Natal, South Africa
Source: JAMA Netw Open. 2019 Nov 1;2(11):e1914378. doi: 10.1001/jamanetworkopen.2019.14378 (PMC6826647; doi:10.1001/jamanetworkopen.2019.14378)
Supplement: Supplement. — eTable 1. Trends in Community Coverage of Selected HIV Prevention and Treatment Programs Overall and by Sex and Age Group eTable 2. Sensitivity Analysis Adjusting for Differences in the Follow-up Times of Participants in the 2016 and 2017 Cohorts [file jamanetwopen-2-e1914378-s001.pdf]

Supplementary Online Content

Kharsany ABM, Cawood C, Lewis L, et al. Trends in HIV prevention, treatment, and incidence in a hyperendemic area of KwaZulu-Natal, South Africa. *JAMA Network Open.* 2019;2(11):e1914378. doi:10.1001/jamanetworkopen.2019.14378

eTable 1. Trends in Community Coverage of Selected HIV Prevention and Treatment Programs Overall and by Sex and Age Group
eTable 2. Sensitivity Analysis Adjusting for Differences in the Follow-up Times of Participants in the 2016 and 2017 Cohorts

This supplementary material has been provided by the authors to give readers additional information about their work.

© 2019 Kharsany ABM et al. *JAMA Network Open*.

eTable 1. Trends in Community Coverage of Selected HIV Prevention and Treatment Programs Overall and by Sex and Age Group

	Men								Women							
	2014 Survey			2015 Survey			% change	P value	2014 Survey			2015 Survey			% change	P value
Age Group	n/N	Weighted % (95% CI)		n/N	Weighted % (95% CI)				n/N	Weighted % (95% CI)		n/N	Weighted % (95% CI)			
**Always used condoms[a,b] with last sex partner among sexually active participants**																
**Overall**	**644/2855**	**24.0**	**(21.5-26.4)**	**728/3275**	**21.6**	**(19.8-23.4)**	**-10%**	**.12**	**1039/5447**	**19.6**	**(17.9-21.3)**	**871/5625**	**16.2**	**(15-17.5)**	**-17%**	**.002**
15-19	102/262	41.1	(32.8-49.5)	159/390	42.4	(36.6-48.3)	3%	.80	109/455	19.8	(15-24.6)	107/522	21.7	(17.6-25.8)	9%	.56
20-24	192/672	32.5	(27.3-37.8)	196/805	24.6	(21-28.1)	-24%	.01	244/1102	24.5	(21.1-27.9)	197/1146	18.2	(15.3-21.1)	-26%	.006
25-29	96/543	18.7	(13.3-24.2)	134/620	21.7	(18.1-25.3)	16%	.37	200/1035	20.7	(17.1-24.3)	166/1142	14.4	(11.9-17)	-30%	.005
30-34	81/429	19.5	(14.5-24.5)	89/531	18.8	(14.7-22.9)	-3%	.84	163/815	19.0	(15-23.1)	138/966	14.7	(12.2-17.2)	-23%	.07
35-39	69/376	18.9	(13.3-24.6)	50/374	12.2	(8.3-16)	-36%	.05	156/734	21.1	(17.5-24.7)	99/718	14.2	(11.1-17.3)	-33%	.004
40-44	63/305	20.1	(14.4-25.9)	59/311	17.0	(12.6-21.4)	-16%	.39	81/633	13.1	(9.4-16.7)	97/627	17.0	(13.5-20.5)	30%	.13
45-49	41/268	15.1	(9.5-20.8)	41/244	15.7	(10.5-20.9)	4%	.89	86/673	13.2	(9.5-16.9)	67/504	14.7	(11.1-18.3)	11%	.57
**Medical male circumcision[c] coverage among all participants**																
**Overall**	**1102/3540**	**31.9**	**(29.5-34.3)**	**1472/3889**	**36.1**	**(34.3-38.0)**	**13%**	**.007**	**1695/4766**	**35.7**	**(33.4-37.9)**	**2519/5207**	**48.2**	**(46.3-50.0)**	**35%**	**<.001**
15-19	317/656	51.4	(46.0-56.8)	485/873	56.6	(52.5-60.7)	10%	.13	202/378	57.4	(51.0-63.8)	314/456	68.8	(64.1-73.4)	20%	.005
20-24	347/813	45.0	(41.1-48.9)	433/900	46.1	(41.9-50.4)	3%	.69	445/962	46.6	(41.9-51.2)	634/1078	58.2	(54.9-61.5)	25%	<.001
25-29	175/601	28.0	(22.8-33.3)	220/636	33.9	(30.0-37.8)	21%	.08	369/942	38.2	(34.0-42.5)	519/1070	48.8	(45.4-52.2)	28%	<.001
30-34	104/460	24.7	(18.7-30.6)	137/540	25.7	(21.3-30.1)	4%	.78	233/715	30.4	(26.0-34.8)	410/885	45.7	(41.4-50.0)	51%	<.001
35-39	81/403	18.1	(12.8-23.5)	101/379	25.8	(21.0-30.7)	42%	.04	191/658	27.2	(22.4-32.0)	287/675	42.8	(38.0-47.6)	57%	<.001
40-44	41/320	11.8	(7.6-16.0)	57/317	16.6	(11.9-21.4)	41%	.13	128/553	21.0	(17.1-25.0)	206/583	36.1	(31.1-41.0)	71%	<.001
45-49	37/287	11.2	(6.5-15.8)	39/244	16.5	(10.6-22.4)	48%	.16	127/558	24.4	(18.8-30.0)	149/460	31.7	(26.0-37.3)	30%	.07
**Knows HIV positive status[a] among all HIV positive participants[d]**																
**Overall**	**504/1014**	**51.8**	**(47.4-56.3)**	**570/922**	**62.9**	**(59.4-66.4)**	**21%**	**<0.001**	**1833/2955**	**64.6**	**(61.9-67.3)**	**2182/2948**	**73.4**	**(71.5-75.4)**	**14%**	**<.001**
15-19	11/36	32.3	(11.2-53.4)	22/39	55.2	(37.2-73.2)	71%	.10	32/131	24.7	(15.7-33.6)	60/133	44.4	(35.1-53.6)	80%	.002
20-24	22/87	26.1	(14-38.1)	23/72	27.8	(15.7-39.8)	7%	.84	184/436	42.5	(36.4-48.7)	185/337	53.5	(47.3-59.7)	26%	.01
25-29	59/171	33.5	(22.7-44.2)	55/130	42.9	(33.6-52.2)	28%	.19	349/578	64.9	(59.1-70.7)	418/606	68.5	(64-73.1)	6%	.33
30-34	99/215	50.5	(42.6-58.3)	124/219	59.7	(52.9-66.5)	18%	.08	395/561	72.8	(68.3-77.4)	528/674	76.9	(73-80.9)	6%	.18
35-39	121/209	60.9	(52.8-69.1)	134/185	71.7	(64.4-79.0)	18%	.05	362/517	75.7	(70.8-80.6)	431/510	84.7	(81.4-87.9)	12%	.003
40-44	116/183	69.8	(62-77.7)	130/174	75.4	(68.5-82.3)	8%	.29	301/426	69.6	(63.9-75.2)	354/431	81.3	(77.1-85.5)	17%	.001
45-49	76/113	61.7	(49-74.4)	82/103	80.6	(71.4-89.7)	31%	.02	210/306	71.3	(64.6-78.1)	206/257	80.1	(74.9-85.4)	12%	.04
**Antiretroviral therapy[a] coverage among all HIV positive participants[d]**																
**Overall**	**341/1014**	**36.7**	**(32.3-41.2)**	**432/922**	**48.6**	**(44.8-52.3)**	**32%**	**<.001**	**1251/2955**	**45.6**	**(42.9-48.2)**	**1743/2948**	**58.8**	**(56.6-60.9)**	**29%**	**<.001**
15-19	9/36	20.9	(5.0-36.8)	19/39	47.4	(29.6-65.2)	127%	.03	23/131	19.3	(10.7-27.9)	46/133	34.8	(25.6-44.0)	80%	.02
20-24	10/87	14.5	(4.6-24.5)	13/72	16.7	(7.1-26.3)	15%	.76	99/436	23.8	(18.5-29.1)	119/337	32.2	(27.0-37.4)	35%	.03
25-29	28/171	16.6	(8.2-24.9)	40/130	30.6	(21.9-39.2)	85%	.02	208/578	40.7	(34.9-46.5)	302/606	50.7	(45.5-55.8)	25%	.01
30-34	59/215	31.4	(23.9-38.9)	82/219	41.4	(34.1-48.7)	32%	.06	270/561	53.1	(48.1-58.1)	419/674	61.3	(56.8-65.9)	15%	.02

© 2019 Kharsany ABM et al. *JAMA Network Open*.

35-39	81/209	46.0	(36.9-55.2)		104/185	56.2	(48.4-64.1)	22%	.09		265/517	56.9	(51.0-62.7)		374/510	72.8	(68.5-77.0)	28%	<.001
40-44	94/183	58.3	(49.1-67.5)		103/174	61.1	(52.2-70.0)	5%	.67		222/426	52.1	(46.7-57.5)		303/431	69.9	(64.7-75.1)	34%	<.001
45-49	60/113	47.4	(35.9-58.8)		71/103	70.1	(59.7-80.4)	48%	.003		164/306	55.8	(48.6-63.0)		180/257	69.2	(63.1-75.3)	24%	.005
**Viral suppression[e,f] among all HIV positive participants[d]**																			
**Overall**	**401/1010**	**41.9**	**(37.1-46.7)**		**456/921**	**50.3**	**(46.8-53.8)**	**20%**	**.005**		**1574/2946**	**54.8**	**(52-57.5)**		**1828/2947**	**61.9**	**(59.7-64.1)**	**13%**	**<.001**
15-19	14/36	39.2	(17.7-60.6)		18/38	42.6	(24.8-60.3)	9%	.81		35/130	27.0	(16.8-37.3)		59/133	46.0	(35.9-56.2)	70%	.009
20-24	15/87	22.6	(9.7-35.4)		15/72	20.4	(9.5-31.4)	-9%	.81		143/433	31.8	(26.1-37.6)		127/337	34.9	(29.3-40.5)	10%	.44
25-29	33/171	15.8	(8.6-23.1)		49/130	37.9	(28.5-47.4)	139%	<.001		278/577	52.7	(47.3-58.1)		327/606	53.5	(48.5-58.6)	2%	.82
30-34	76/215	39.1	(29.9-48.3)		91/219	44.7	(37.1-52.4)	14%	.35		313/561	56.9	(51.7-62.1)		430/674	63.6	(59.2-67.9)	12%	.05
35-39	92/208	47.0	(38.3-55.7)		107/185	57.0	(48.9-65)	21%	.10		328/513	67.5	(61.6-73.4)		384/509	74.8	(70.6-79.1)	11%	.05
40-44	100/180	62.5	(53.8-71.1)		105/174	60.2	(52.4-68.1)	-4%	.71		273/426	63.5	(57.6-69.4)		311/431	70.9	(65.8-76)	12%	.06
45-49	71/113	64.0	(51.9-76.2)		71/103	68.0	(57.4-78.6)	6%	.63		204/306	67.0	(60.7-73.4)		190/257	76.0	(70.2-81.7)	13%	.04
**Progress towards UNAIDs 90-90-90 composite target of 73% of all HIV positive individuals on treatment achieving viral suppression[g]**																			
**Overall**	**284/1010**	**30.8**	**(26.9-34.8)**		**376/921**	**42.6**	**(38.9-46.3)**	**38%**	**<.001**		**1086/2946**	**39.9**	**(37.2-42.5)**		**1545/2947**	**52.5**	**(50.3-54.7)**	**32%**	**<.001**
15-19	7/36	17.2	(2.1-32.3)		14/38	35.3	(18.3-52.2)	105%	.12		20/130	14.4	(7.8-21)		37/133	27.2	(18.3-36.1)	89%	.02
20-24	8/87	11.5	(2.1-21)		9/72	11.4	(3.3-19.5)	-1%	.98		73/433	17.5	(13.1-22)		94/337	25.3	(20.6-30)	44%	.02
25-29	21/171	12.2	(5.1-19.4)		32/130	25.6	(17.3-33.9)	109%	.02		183/577	37.2	(31.5-42.9)		254/606	43.2	(38-48.3)	16%	.12
30-34	51/215	27.3	(20.2-34.4)		73/219	37.3	(30-44.6)	37%	.05		234/561	44.9	(40.2-49.7)		371/674	54.9	(50.5-59.3)	22%	.003
35-39	64/208	35.0	(26.2-43.8)		92/185	49.8	(42-57.6)	42%	.01		229/513	50.5	(44.6-56.4)		345/509	67.9	(63.4-72.4)	34%	<.001
40-44	79/180	53.0	(43.8-62.1)		91/174	53.3	(44.7-61.8)	1%	.96		203/426	48.0	(42.6-53.5)		277/431	63.9	(58.5-69.2)	33%	<.001
45-49	54/113	42.9	(31.7-54.1)		65/103	64.5	(53.9-75.1)	50%	.005		144/306	49.7	(42.5-56.9)		167/257	64.5	(58.4-70.6)	30%	.002

a=self-report; **b**=men reporting condom use and women reporting partner using condoms; **c**=men reported being medically circumcised and women reporting partner medically circumcised; **d**=laboratory confirmed HIV positive status; **e**=viral suppression defined as viral load <400 copies per mL; **f**=Four men and nine women were missing viral load data in the 2014 Survey and one man and one woman were missing viral load data in 2015 Survey, percentages exclude missing viral load data; **g**= composite measure of UNAIDS 90-90-90 targets of diagnosis, treatment coverage and viral suppression - 90% of all HIV positive people diagnosed (90%). 90% of those diagnosed on treatment (81%) and 90% of those treated to have achieved viral suppression (73%) - resulting in 73% of all HIV positive people diagnosed on treatment ie antiretroviral therapy (ART) achieving viral suppression.

© 2019 Kharsany ABM et al. *JAMA Network Open*.

eTable 2. Sensitivity Analysis Adjusting for Differences in the Follow-up Times of Participants in the 2016 and 2017 Cohorts[a,b]										
	**2016 Cohort**			**2017 Cohort**			**Relative change in HIV incidence**			
	**HIV seroconversions n / total N**	**Person-Years[b]**	**Incidence Rate per 100 person-years (95% CI)**	**HIV seroconversions n / total N**	**Person-years[b]**	**Incidence Rate per 100 person-years (95% CI)**	**Incidence Rate Ratio (95% CI)**	**P value**	**Adjusted[d] Incidence Rate Ratio (95% CI)**	**P value**
**Men**										
**Overall**	**39/1415**	**2236**	**1.64 (1.06-2.53)**	**31/1829**	**2560**	**1.32 (0.90-1.92**	**0.80 (0.45-1.44)**	**.46**	**0.83 (0.46- 1.47)**	**.51**
15-19	4/480	749	0.45 (0.15-1.31)	3/668	940	0.24 (0.08-0.74	0.53 (0.11-2.52)	.43	0.53 (0.11- 2.56)	.43
20-24	16/482	760	1.61 (0.87-2.98)	11/572	800	1.18 (0.61-2.28)	0.73 (0.30-1.81)	.50	0.78 (0.31-1.92)	.58
25-29	13/263	423	2.90 (1.41-5.95)	13/339	467	2.84 (1.56-5.14)	0.98 (0.38-2.49)	.96	1.08 (0.40-2.90)	.88
30-35	6/190	304	2.27 (0.79-6.49)	4/250	354	1.45 (0.52-4.05	0.64 (0.15-2.78)	.55	0.65 (0.16-2.75)	.56
**Women**										
**Overall**	**124/1820**	**2815**	**3.72 (2.92-4.74)**	**84/2078**	**2887**	**2.8 (2.22-3.53)**	**0.75 (0.54-1.05)**	**.10**	**0.77 (0.55-1.09)**	**.14**
15-19	51/623	954	4.91 (3.49-6.91)	30/743	1032	2.74 (1.84-4.09)	0.56 (0.33-0.95)	**.03**	**0.54 (0.32-0.92)**	**.02**
20-24	41/587	904	4.32 (2.98-6.31)	41/668	923	4.26 (3.04-5.97)	0.99 (0.59-1.63)	.95	1.00 (0.60-1.68)	.99
25-29	26/389	611	2.55 (1.59-4.07)	10/416	580	1.87 (0.93-3.77)	0.73 (0.32-1.71)	.48	0.77 (0.33-1.79)	.55
30-35	6/221	347	0.71 (0.30-1.64)	3/251	352	0.47 (0.15-1.51)	0.66 (0.16-2.81)	.58	0.72 (0.17-3.03)	.65
**Overall**	**163/3235**	**5052**	**2.55 (2.01-3.23)**	**115/3907**	**5447**	**1.96 (1.62-2.37)**	**0.77 (0.57-1.04)**	**.09**	**0.80 (0.59-1.10)**	**.17**

a=There were 278 Western blots confirmed HIV seroconvertors; 163 and 115 in the 2016 and 2017 Cohorts, respectively. This excluded the one blot-unconfirmed positive ELISA result and 9 ELISA seroconvertors who were found to be HIV RNA positive at enrolment. HIV incidence rates and 95% confidence intervals were weighted to account for the multi-level sampling design and estimated using survey Poisson regression models and reported as per 100- person years.
b=shortened follow-up time to match for comparable person years of follow-up
c=adjusted for sex, age, education, lifetime number of sex partners and HIV testing history

© 2019 Kharsany ABM et al. *JAMA Network Open*.
